# Microbial Musings – December 2021

**DOI:** 10.1099/mic.0.001141

**Published:** 2022-01-31

**Authors:** Gavin H. Thomas

**Affiliations:** ^1^​ Department of Biology, University of York, York, UK

We close this year with a bumper issue of the journal and too many articles to write full stories about. This is a tremendous way to end 2021and really shows that the #publishingforthecommunity message is getting out. Hats off to Ruth Massey (@ProfRuthMassey) for being our most published author of 2021, with Andrew Edwards a close second (@bugsinblood), following on from Cynthia Whitchurch (@Cwhitch) in 2020, all role models for young microbiologists in how to balance their portfolio of publications to include Society journals.

We start with our latest Microbe Profile, which is a bacterium, *Buchnera aphidicola *, that might not be very well known to many of you, but one that I know very well. In the article, written by insect symbiosis guru Nancy Moran (@NancyMoran15), from the University of Texas at Austin, USA, she introduces this obligate symbiont of the pea aphid, which has so many interesting features about its biology that it is difficult to know where to start! The relationship of the bacterium and its host is ancient, over 100 million years old, and remarkably the bacteria live inside special cells called bacteriocytes that the aphid has evolved to house them. The reason the aphid goes to all this trouble is that it lives on a sugary diet of plant phloem sap, which is lacking in most essential amino acids. During rapid growth of the embryos in the aphid they use the symbiont to upgrade nitrogen from waste products into essential amino acids, some fascinating research I was involved with directly when working with Angela Douglas of Cornell University, USA, when she was in York [1, 2]. The genome sequence of this bacterium is also highly reduced at 0.64 Mb, which was figured out by Shuji Shigenobu and colleagues at the University of Tokyo, Japan, in a landmark paper in 2000 after he had dissected bacteria from 2000 pea aphids to get enough DNA to sequence [3]! Its tiny genome is essentially a subset of the *Escherichia coli* genome, which is why I ended up working with Angela on it, and we did some nice work building a whole-genome metabolic reconstruction, which was really complete, as all the metabolic genes in *B. aphidicola* had characterized orthologues in *E. coli* [4]. However, while bioinformatically a model system, experimentally the bacterium cannot be cultured, and there are outstanding questions as to how it exchanges nutrients with the host, as its genome lacks obvious transporters for most things. I suggest you read Nancy’s Profile and you might get hooked by this bacterium like I did!

Sticking with microbes living in unusual associations with animals, we move on to a paper from the group of Mathew Upton (@mat_upton) at the University of Plymouth, UK, who has been isolating bacteria from the microbiomes of deep sea sponges. The paper, which includes authors Poppy Hesketh-Best (@PoppyBest3), Philip Warburton (@DrPhilWarburton) and Kerry Howell (@DeepSeaEcol), led by Matthew Koch, describes the isolation of multiple Proteobacteria, Actinobacteria and Firmicutes from two previously uncharacterized species of hexactinellid sponges [5]. There was large diversity across the two sponges, the profile of which changed when they were cultured at increased pressure of 5 bar, which is the same as ~50 m deep, when they then isolated species of *Microbacterium* and an ‘uncultured bacterial clone’ that didn’t grow at atmospheric pressure. Taking their large library of over 1000 isolates that they had collected, they then screened for antimicrobial activity against *Micrococcus luteus *, *Staphylococcus aureus* and *E. coli *, and found ~20 with activity and some were active against both Gram –ve and Gram +ve bacteria. Marine microbes have huge potential to provide new natural products with antimicrobial activity [6], such as anthracimycin isolated from a marine actinomycete that is active against MRSA [7], and this article is part of our Marine Microbiology collection that was edited by Kate Duncan (@PoppyBest3) and Alex Chase (@microbomics) to highlight the microbiological potential of this enormous global habitat.

The use of bacteriophage as a therapeutic has been touted since its co-discovery by Félix d’Hérelle just over 100 years ago, and he always had applications in mind for his 'bacterial eaters'. While then taking the backstage to antibiotics through much of the 20th century, with limited topical use in some countries, there is now revived interest and more extensive clinical use of phage [8], for example in the study I mentioned in the September Musings in relation to Graham Hatfull’s (@GHatfull) research with clinicians in London, UK, for producing phage cocktails to treat cystic fibrosis patients infected with *Mycobacterium abscessus* [9]. In this issue two related papers make important contributions to the conceptual and practical applications of combinations of phage for use in clinical settings and give us a deeper understanding of how to assemble simple and effective phage cocktails.

The first of these papers was the second to use our new Rapid Review track and was a large study from the group of Adam Arkin (@AdamArkinLab) at the University of California, Berkeley, USA [10]. The study was an attempt to take a systematic approach to understand the genetic basis of susceptibility of *Salmonella enterica* subsp. Typhimurium to a panel of 11 diverse lytic bacteriophage and from this also examine how the bacteria confer cross-resistance to multiple phage. In the work, which was led by Ben Adler (@ben_a_adler), with multiple colleagues, including Alexey Kazakov (@AlexeyKazakov8), Hualan Liu (@lanlanliu2), Heloise Carion (@CarionHeloise), Adam Deutschbauer (@DeutschbauerLab) and Vivek Mutalik (@vivek_mutalik), the authors first assembled a diverse set of phage from five of the nine major phage families, including a number from therapeutic phage cocktails. To identify genes that confer resistance to the phage they used a random barcoded transposon sequencing method (RB-TnSeq) they had developed previously [11, 12] to create libraries of Tn-mutants for a *S*. Typhimurium strain derived from LT2. Populations of bacteria representing the libraries were then exposed to each of the phage, in liquid or on plates, and they compared which genes were required for survival by sequencing the barcodes of surviving bacteria and looking for enriched genes, who's disruption correlated with phage resistance. Having performed 42 different variants of this technique with their library of phage, they identified just over 300 phage–host gene interactions. They found, of course, that many of the enriched genes are involved in receptor function at the cell surface, as this is the first essential step of the lytic cycle and total loss of LPS confers resistance to many of the phage. Picking out one nice example, as it relates to some of my research in York with Marjan van der Woude, they found for the T5-like phage Aji_GE that mutations in *oafA* confered high fitness, and this gene encodes a protein we have helped to characterize as a membrane-bound O-acetyltransferase [13]. This presumably suggests that this phage needs the specific O-acetylation of the abequose sugar on LPS (the O5 serotype) to bind, and it is known that O-acetylation of the O-antigen repeat can alter phage sensitivity directly, as the O-acetylation of the rhamnose sugar in LPS confers resistance to lysis by BTP1 phage [14]. There is a wealth of other interesting information in the paper mapping phage resistance to changes in receptor-based phenotypes. The authors then go on to investigate genes that are associated with resistance across multiple phage types, including genes encoding functions clearly not directly related to receptor function. After examining some commonly induced genes, and investigating the role of some of these by further RNAseq experiments, they argue that elevated RpoS activity could be a common factor that can confer cross-resistance, which is sometimes related to the upstream function of RpoN. Finally, they took a collection of 21 diverse *S*. Typhimurium strains and then related their known genotype to their ability to be infected by the 11 phage, again finding many interesting patterns. For example, one strain with a mutation in *oafA* is resistant to the Aji_GE phage, which nicely confirmed their earlier data. Overall, the paper provides a feast of information and importantly could be used to consider the use of mixes of phage where the bacterial cell cannot confer cross-resistance and so help in the design of improved phage cocktails.

Thinking about how much complexity to put into the cocktail is the topic of our second related paper from Rosanna Wright (@rctwright) when at the University of York, UK, with my colleagues Ville Friman (@FrimanScience) and Maggie Smith (@MaggieCMSmith) working with *Microbiology* senior editor Mike Brockhurst (@BrockhurstLab) at the University of Manchester, UK [15]. In this work they use a biodiversity–ecosystem function relationship analysis to study how species richness, here represented by the bacteriophage, impacts on the efficacy of the phage cocktail being used. In their work they use a panel of 12 lytic phage specific for *Pseudomonas aeruginosa* and design experiments to combine these at increasing levels of complexity, measuring bacterial load after 24 h as their measure of efficacy. Their conclusions are logical and clear [15]: (1) there are increasingly diminishing returns from adding additional phage to the cocktail in terms of efficacy and (2) increasing the diversity of the phage, in terms of their receptor mechanisms, improves efficacy. While they only varied phage by the receptor types they used, they point out that including phage with different abilities to tolerate various anti-phage mechanisms would also improve the efficacy of the mix. Together, these two papers provide important theoretical and practical guidance and insights into how to prepare better phage therapy cocktails.

Our next article concerns a poorly characterized family of ancient bacterial membrane proteins called DedA proteins. One of the best experimental systems for studying DedA proteins is in *E *. *coli *, which has eight of them scattered around the genome. In an landmark study from the group of William Doerrler (@DoerrlerWilliam) at Louisiana State University, USA, the authors have been able to find phenotypes for a number of systems after complementing an octuple mutant, which in itself was non-viable at high temperature and failed to divide normally [16]. One of those eight proteins was YqjA, which the paper in this month’s provides the first experimentally derived membrane topology of a DedA protein. The work, from the group of Chris Mulligan (@Chris_mulligan) at the University of Kent, UK, combined new methods of predicting protein structure by evolutionary covariance with the experimental testing of this using topological mapping [17]. The experimental method requires first removing all of the codons for cysteine from the gene, and then making versions with single cysteine codons added back in. This enabled them to use a thiol-reactive dye that can only label parts of the protein on the outside of the inner membrane, compared to another that can penetrate the membrane, to then work out the position of the labelled cysteine in the protein. Some of the single cysteine mutants cross-link to each other, which the authors suggest supports a model for parallel dimer formation in the native membrane. Using Lucy Forrest’s (@LucyRForrest) AlignMe tool [18], they argue that the topology of YqjA is likely representative of the entire family. While the physiological function of YqjA and other DedA-family proteins is still not completely clear, the experimentally supported structural model is consistent with one proposed function as a monovalent cation/H^+^ exchanger, although clearly there is a lot more to learn about these novel transporter family.

We end this month with something completely different. My colleague in the world of bacterial sialobiology, Graham Stafford (@GrahamPStafford) from the University of Sheffield, UK, has been working on a new project to understand the physiology of bacteria isolated from ‘fatbergs’ [19]. These enormous collections of fats, oils and grease (FOG) are increasingly blocking up sewage pipes and have been featured numerous times in the news of late. In fact, a neighbour of mine’s basement kitchen flooded with sewage due to a small fatberg that was traced the local fast food restaurant, so I can assure you that this can be a real problem! In this study from Elizabeth Court and others from the Stafford lab, with collaborators Roy Chaudhuri (@RoyChaudhuri) and Jags Pandhal (@JPandhal), also at Sheffield, the group first got their hands dirty with some real fatbergs, courtesy of Thames Water. They were able to first determine that palmitic acid was the most abundant fatty acid, at around 75 % of the total, and using enrichment culture coupled to a plate assay for lipase activity, they isolated five lipolytic species to study further. These were all Gram-negative and were strains of *Serratia marcescens *, *Serratia liquefaciens *, *Klebsiella oxytoca *, *Klebsiella pneumoniae* and *Acinetobacter bouvetii *. Each was equipped with secreted lipases of different specificity and fatty acid transport and catabolism genes. Finally, they performed some initial microbiome analysis on the fatberg and found quite wide diversity at each site, although they consistently found *Xanthomonas* and *Rhodobacter *, species known to degrade lipids, and an anaerobe that is new to me called *Phascolarcobacterium* (quickly checks his Bergey’s – a propionate-fermenting Clostridiales). They also found isolates from the species that they had just characterized, as expected. Of course, they end by speculating about how this knowledge might be used to help either prevent fatberg formation or treat fatbergs themselves, and acknowledge that as the fatberg contains other materials such as wet wipes, etc., they might need a complex consortium of microbes to do this.

This issue also contains two reviews that are worth checking out. The first, from Megan Sloan and colleagues at the University of Glasgow, UK, is about the uptake and storage of metals in apicomplexan parasites, and will feature in our Metals and Microbes collection [20]. This was a fascinating review and I found myself contrasting its revelations with what I know about how bacteria solve similar problems in the uptake, efflux and storage of metals such as iron, zinc and copper, which of course in these organisms is even more complex as they also have to be moved between different organelles. The second, from Yue Yuan On and *Microbiology* Senior Editor Martin Welch, comprises an analysis of DNA mismatch repair (MMR) machinery in the human pathogen *P. aeruginosa *, which they argue differs significantly from that of its relative *E. coli* and is very important for the evolution of this pathogen *in vivo* – a subject that demands further study [21].

This article marks the end of 2 years of *Microbial Musings* and for 2022 there is going to be a bit of a change, as we will be featuring a series of articles about aspects of the history of the journal, including three pieces by historian of science Peter Collins, who has been working hard studying the archives of the Society – his first article on the origins of the journal will appear in the January issue. Hence, the *Musings* will be back in February and will be every other month through this coming year.

Finally, on a sadder note, we have heard that former Editor-in-Chief of the journal Derek Smith, who led the *Journal of General Microbiology* (JGM) through the late 1980s, recently passed away. There will be a full obituary of Professor Smith on the society website in early 2022.

## References

[1] Macdonald SJ, Thomas GH, Douglas AE (2013). Waste not, want not: Nitrogen recycling by metabolic pathways shared between an animal and its symbiotic bacteria. Biochem.

[2] Wilson ACC, Ashton PD, Calevro F, Charles H, Colella S (2010). Genomic insight into the amino acid relations of the pea aphid, *Acyrthosiphon pisum*, with its symbiotic bacterium *Buchnera aphidicola*. Insect Mol Biol.

[3] Shigenobu S, Watanabe H, Hattori M, Sakaki Y, Ishikawa H (2000). Genome sequence of the endocellular bacterial symbiont of aphids *Buchnera* sp. APS. Nature.

[4] Thomas GH, Zucker J, Macdonald SJ, Sorokin A, Goryanin I (2009). A fragile metabolic network adapted for cooperation in the symbiotic bacterium Buchnera aphidicola. BMC Syst Biol.

[5] Koch MJ, Hesketh-Best PJ, Smerdon G, Warburton PJ, Howell K (2021). Impact of growth media and pressure on the diversity and antimicrobial activity of isolates from two species of hexactinellid sponge. Microbiology.

[6] Jensen PR, Fenical W (1996). Marine bacterial diversity as a resource for novel microbial products. J Ind Microbiol Biotechnol.

[7] Hensler ME, Jang KH, Thienphrapa W, Vuong L, Tran DN (2014). Anthracimycin activity against contemporary methicillin-resistant *Staphylococcus aureus*. J Antibiot.

[8] Levin BR, Bull JJ (2004). Population and evolutionary dynamics of phage therapy. Nat Rev Microbiol.

[9] Dedrick RM, Guerrero-Bustamante CA, Garlena RA, Russell DA, Ford K (2019). Engineered bacteriophages for treatment of a patient with a disseminated drug-resistant *Mycobacterium abscessus*. Nat Med.

[10] Adler BA, Kazakov AE, Zhong C, Liu H, Kutter E (2021). The genetic basis of phage susceptibility, cross-resistance and host-range in *Salmonella*. Microbiology.

[11] Price MN, Wetmore KM, Waters RJ, Callaghan M, Ray J (2018). Mutant phenotypes for thousands of bacterial genes of unknown function. Nature.

[12] Wetmore KM, Price MN, Waters RJ, Lamson JS, He J (2015). Rapid quantification of mutant fitness in diverse bacteria by sequencing randomly bar-coded transposons. mBio.

[13] Pearson CR, Tindall SN, Herman R, Jenkins HT, Bateman A (2020). Acetylation of surface carbohydrates in bacterial pathogens requires coordinated action of a two-domain membrane-bound acyltransferase. mBio.

[14] Kintz E, Davies MR, Hammarlöf DL, Canals R, Hinton JCD (2015). A BTP1 prophage gene present in invasive non-typhoidal *Salmonella* determines composition and length of the O-antigen of the lipopolysaccharide. Mol Microbiol.

[15] Wright RCT, Friman V-P, Smith MCM, Brockhurst MA (2021). Functional diversity increases the efficacy of phage combinations. Microbiology.

[16] Boughner LA, Doerrler WT (2012). Multiple deletions reveal the essentiality of the *DedA* membrane protein family in *Escherichia coli*. Microbiology (Reading).

[17] Scarsbrook HL, Urban R, Streather BR, Moores A, Mulligan C (2021). Topological analysis of a bacterial DedA protein associated with alkaline tolerance and antimicrobial resistance. Microbiology.

[18] Stamm M, Staritzbichler R, Khafizov K, Forrest LR (2013). Alignment of helical membrane protein sequences using AlignMe. PLoS One.

[19] Court EK, Chaudhuri RR, Kapoore RV, Villa RX, Pandhal J (2021). Looking through the FOG: microbiome characterization and lipolytic bacteria isolation from a fatberg site. Microbiology.

[20] Sloan MA, Aghabi D, Harding CR (2021). Orchestrating a heist: uptake and storage of metals by apicomplexan parasites. Microbiology.

[21] On YY, Welch M (2021). The methylation-independent mismatch repair machinery in *Pseudomonas aeruginosa*. Microbiology.

